# Probable Mechanism of Antiepileptic Effect of the Vagus Nerve Stimulation in the Context of the Recent Results in Sleep Research

**DOI:** 10.3389/fnins.2020.00160

**Published:** 2020-02-28

**Authors:** Ivan N. Pigarev, Marina L. Pigareva, Ekaterina V. Levichkina

**Affiliations:** ^1^Institute for Information Transmission Problems (Kharkevich Institute), Russian Academy of Sciences, Moscow, Russia; ^2^Institute of Higher Nervous Activity and Neurophysiology, Russian Academy of Sciences, Moscow, Russia; ^3^Department of Optometry and Vision Sciences, The University of Melbourne, Parkville, VIC, Australia

**Keywords:** epilepsy, vagus nerve, electric stimulation, antiepileptic effect, visceral theory of sleep

The goal of this article is to discuss the possible contribution to antiepileptic effects of the vagus nerve stimulation (VNS) from the functional connectivity between the cortex and internal organs. According to our previous work, this connectivity is particularly prominent during sleep, the brain state when epileptic activity is prominent, as well. As such, the relationship between the brain and the viscera needs to be put into the equation when considering VNS as a treatment for epilepsy.

Vagus nerve stimulation is widely used as a seizure-preventive action in many types of otherwise incurable epilepsy and is extensively studied for treating other conditions ranging from rheumatoid arthritis to depression (Vonck et al., 2001; Groves and Brown, 2005; Yuan and Silberstein, 2016; Dibue-Adjei et al., 2019; Noller et al., 2019). It is well-known that vagus nerve is engaged in the bidirectional information transfer between the internal organs and the brain, but how changes in activity going along visceral pathways may be related to paroxysmal events occurring in various brain areas remained unclear. The available literature describe several ideas proposed to explain possible seizure preventing action of VNS, which mainly based on molecular mechanisms of synaptic transmission in the central nervous system. Although neuronal desynchronization, hippocampal plasticity, anti-inflammatory immune changes, and changes in neurotransmitter concentrations are all currently considered as possibly involved in its antiepileptic effects (Yuan and Silberstein, 2016), none of the existing theories explains the impressive variety of demonstrated effects of VNS. We are offering for discussion another suggestion, based on the role of the vagus nerve in autonomic regulation and on the recent results of sleep studies.

In this opinion article we do not present any new experimental results, but only aim to provide a possible link between four seemingly unrelated clusters of well-established physiological observations, which, being considered together, might offer new directions for thinking and investigation of VNS mechanisms.

First, there is a well-established connection between epileptic seizures and the state of sleep (e.g., Shouse et al., 1996; Herman et al., 2001; Dinner, 2002; Combi et al., 2004; Pavlova et al., 2004; Durazzo et al., 2008; Hofstra and de Weerd, 2009; Kothare and Kaleyias, 2010; Mirzoev et al., 2012). Ictal activity is generally most frequent in slow-wave sleep and during transition from wakefulness to sleep, but is very rarely present in REM-sleep. Approximately half of all recorded seizures are happening during slow wave sleep while this state occupies less than one third of circadian cycle in humans. In addition, it is highly likely that some seizures happening during sleep may stay undetected. We group these observations in the first cluster of the data.

A second cluster of observations to consider involves another generally recognized feature of many types of epilepsy—the epileptogenic effects of rhythmic stimulations delivered to various sensory systems (see e.g., Kaplan, 2003; Guerrini and Genton, 2004; Hirsch et al., 2004; Michelucci et al., 2004; Wilkins et al., 2004; Parra et al., 2005). Ictal activity provoked by rhythmic exteroceptive stimulation may have similar mechanism to physical resonance systems. Theoretically, any circuit with positive feedback has its own internal resonance frequency. Rhythmic external stimulation, even relatively weak, would initiate strong oscillations if the frequency of this stimulation approaches this resonance frequency. In the nervous system, it would manifest as paroxysmal activity. Neuronal circuits with feedback are common features at all levels of the nervous system, and resonance effects in the nervous system were indeed demonstrated (see e.g., Hutcheon and Yarom, 2000; Herrmann, 2001). In addition, widely accepted mechanism of pathologically elevated excitability in epileptic focus can be a part of this mechanism as it would be able to increase probability of the weak rhythmic afferent signal to reach thresholds for such resonant oscillations.

Resonant model of epileptogenesis implies the presence of two components. The first component is the local neuronal network with positive feedback, which has the fundamental frequency of oscillation and is susceptible to paroxysmal activity. The second component is the rhythmic afferent flow directed to that network that may cause the resonant activation. Neither of these two components can provoke an ictal activity alone.

Anticonvulsant drugs can elevate the activation thresholds of the resonant network, diminishing responses to the afferent signals, but are not able to eliminate the incoming signals driving the network into ictal activity.

It might seem that resonance evoked by afferent inputs cannot be a mechanism of the previously mentioned epileptic activity during sleep. In classical neuroscience paradigm, sleep is considered as the state when brain is sensory deprived and any external rhythmic stimulation is excluded. However, recent sleep studies offer an alternative source of rhythmic sensory afferent signals directed to cerebral cortex, that are not attenuated, but likely to be enhanced during sleep. We previously demonstrated that during sleep cortical sensory areas begin receiving information coming from various visceral organs (Pigarev, 1994, 2013; Pigarev et al., 2013; Pigarev and Pigareva, 2014, 2018). Experiments that demonstrated propagation of the visceral afferent signals to the cerebral cortex during sleep, were performed on gastrointestinal and cardio-respiratory systems, and their work is inherently rhythmical (Pigarev, 1994; Pigarev et al., 2013; Lavrova, 2019; Lavrova et al., 2019). Thus, nervous signals in the involved sensory pathways would be rhythmically organized during sleep. These results of sleep studies comprise the third block of the relevant observations.

Observable rhythmic motility of the visceral organs generally has relatively low frequencies in comparison to frequencies of exteroceptive sensory stimulation reported as epileptogenic (10–50 Hz). However, nervous signals from these organs transferring along the nerves might have another organization in time, and more complicated frequency spectrums. Namely, these nervous signals can interfere with resonant frequencies of different brain regions leading to ictal events. In addition to that, one should remember that not only the exact correspondence of frequencies leads to a resonance, as resonance is possible for both the fundamental frequency and for its harmonics and sub harmonics. Afferent information flow from some internal organs may have frequency pattern close to the resonant frequency of a particular brain circuit susceptible to paroxysmal events. In our opinion, during sleep epileptic activity could be initiated in such area by the rhythmical visceral afferentation, similarly to generation of such events by rhythmical exterosensory stimulation in wakefulness. Indeed, registration of vagal electrical activity during natural sleep in cat demonstrated synchronized appearance of spindle-like activity in vagus itself and in a range of cortical and subcortical regions receiving vagal input (Leichnetz, 1972).

Assuming that in some cases epileptic events are generated in response to resonant frequencies of visceral afferentation, antiepileptic effect of VNS may have a simple explanation. For therapeutic purpose, stimulation usually is applied to the left cervical vagal trunk that contains fibers from the recurrent laryngeal, cardiopulmonary, and subdiaphragmatic vagal branches. At this level, roughly 80% of the vagal fibers are afferent, and 20% are efferent (Krahl, 2012). With such fiber composition, VNS would change the pattern of visceral activity transmitted to the brain by the vagus nerve, and is likely to cause prominent reorganization of activities within the crucial structures receiving vagal afferentation and altering further visceral input, such as nucleus tractus solitarius, parabrachial nucleus, and hypothalamus.

The role of VNS as disrupting the afferent flow to the regions susceptible for convulsive activity is in good accordance with the ability of a surprisingly wide range of frequencies of VNS to reduce epileptic activity. Frequencies from 1 to 143 Hz were used for this purpose, although frequencies above 50 Hz are not recommended in clinical practice as potentially damaging to the vagus nerve itself (for details see Terry, 2014). It was also proposed that stimulation of the afferent vagus nerve fibers can change the fundamental resonant frequencies of the brain circuits itself (Fanselow, 2012).

Furthermore, stimulation of the efferent vagal fibers also alters the frequencies of rhythmically working visceral organs, such as heart, stomach and intestine (e.g., Martinson, 1965; Chang et al., 2003; Osharina et al., 2006; Tong et al., 2010; Bonaz et al., 2016; Frøkjaer et al., 2016). Changes of rhythmicity of the various visceral organs elicited by stimulation of the vagus efferent fibers and altering activity of the visceral organs should modify the frequency composition of the visceral afferent signals coming to the brain areas not only by vagal, but also by spinal cord pathways. All of the changes described above are expected to move visceral afferent frequencies out of the resonance range, thereby blocking paroxysmal activity. These visceral effects of VNS we present as the fourth cluster of the relevant observations.

Taking all the above mentioned into account it seems important to study background spike firing in the vagus nerve during wakefulness and sleep, and the effect of VNS on this firing. The former subject was actually investigated in one study in cat. It was shown that during natural sleep activity in vagus nerve itself and in a range of cortical and subcortical regions receiving vagal input demonstrated spindle-like synchronized pattern, and prominent amplitude and frequency differences were noted between wakefulness, slow wave and REM sleep states (Leichnetz, 1972). However, the technique used at that time (ink electroencephalography) did not allow observing single spikes and only the integrated power of spike activity was recorded. Nevertheless, the results obtained by Leichnetz revealed that circadian dynamic is indeed present in vagal activity. Ramet et al. (1992) also indirectly observed increased vagal activity during sleep in humans. However, to the best of our knowledge, this topic has not been studied in detail using contemporary techniques. Such studies would be instrumental in finding the optimal parameters of VNS.

This opinion may meet disagreement based on a doubt concerning the increased involvement of the cerebral cortex in the processing of visceral information during sleep. Our view is based on electrophysiological experiments performed in rabbits, cats, and monkeys (see for review, e.g., Pigarev, 2014; Pigarev and Pigareva, 2014). However, results of these studies are not widely known yet, most likely because their subject, being located between three very different disciplines—classical sensory physiology, physiology of the visceral systems and sleep research, usually slips attention of the corresponding three groups of researchers. Recently several independent laboratories started demonstrating similar results. Lecci et al. (2017) found the relationship between slow periodicity in the cortical EEG during sleep and heart rate variability. In experiments combining functional MRI and electrogastroscopy the reflection of slow gastric rhythms in cortical sensory areas was observed in humans (Rebollo et al., 2018). There is also a growing body of evidence pointing to the link between visceral abnormalities and psychiatric disorders. For example, it was proposed that degeneration of cells in the intestinal enteric nervous system might have causal link with the following appearance of Parkinson disease (for a review see e.g., Smith and Parr-Brownlie, 2019). Fatal familiar insomnia syndrome, which leads to progressive inability to sleep, also results in severe autonomic dysfunction finally finishing by death (Lugaresi and Provini, 2007). It is generally believed that insular, orbitofrontal and medial prefrontal areas are directly involved in autonomic regulation (Neafsey, 1990; Ongür et al., 1998; Ongür and Price, 2000; Nieuwenhuys, 2012), but at the same time they are known to take part in regulation of the sleep-wake cycle (Saper et al., 2010; Chen et al., 2016). Significant increase in neuronal activity associated with slow waves during sleep was found in the inferior frontal, medial prefrontal, posterior cingulate areas and the precuneus (Dang-Vu et al., 2008). The overall, it was found that reorganization of the interneuronal connections during wake to sleep transition leads to formation of new cortical neuronal networks (Larson-Prior et al., 2011).

One may argue that VNS is also efficient in wakefulness. Influence of VNS in wakefulness can be understood taking into account that seizures often start in the high order associative cortical areas. It is known that local or partial sleep also starts developing from these cortical areas (Pigarev et al., 1997). According to the visceral theory of sleep (Pigarev and Pigareva, 2014) development of the local sleep in limited parts of the cerebral cortex indicates the onset of visceral information transfer to those cortical areas while behaviorally this state correspond to wakefulness or drowsiness. In addition, it was reported that epileptic attacks often happen during developing drowsiness (Mirzoev et al., 2012).

On the other hand, some cortical areas receiving vagal input, such as the insular cortex, are involved in the processing of visceral information in wakefulness as well. The role of the insular cortex in mediating bodily feelings—“interoceptive awareness”—has been discussed by Craig (Craig, 2011; for a review of the insula functions see Nieuwenhuys, 2012). Thus, rhythmic visceral afferentation definitely reaches insular cortex in wakefulness, and “visceral” mechanism of epileptogenesis may work through the insular network not only during sleep. However, we have recently reported the prevalence of insular neurons responding to non-noxious intestinal electrostimulation in slow wave sleep in comparison to wakefulness (Levichkina and Pigarev, 2016), and it is therefore expected that responses of the insular cortex to VNS can be more prominent in sleep as well.

Finally, it was hypothesized (Morchiladze et al., 2018) that some mental disorders can be associated with pathological chronic inactivation of the mechanisms blocking the propagation of visceral information toward the central nervous system in wakefulness. As a result, these visceral signals could be added to the normal exterosensory information flows as noise, disrupting their normal analysis. If this “noise” has rhythmic structure, it would be able to evoke seizers in a similar way to the exterosensory rhythmic signals.

In the context of the probable role of the visceral rhythmic afferentation in genesis of paroxysmal events it might be important to analyze the noted comorbidity of epilepsy to a number of visceral issues such as gastrointestinal bleed, chronic diseases of cardio- and respiratory systems, pneumonia and diabetes (Gaitatzis et al., 2004). It is not excluded that described positive effect of the ketogenic diet for treatment of epilepsy (e.g., D'Andrea Meira et al., 2019) also can be related to probable change of some rhythms in gastro-intestinal system and consequently of frequencies in the visceral afferent messages in response to the changed food content.

We do not intend to present this “visceral” mechanism of seizure generation and proposed mechanism of VNS antiepileptic effect as the only possibility. Obviously different types of epilepsy are likely to have other mechanisms of seizure initiation. The goal of our comment is to draw attention to the additional factor, which has not been considered yet. Important and unexpected feature of the proposed mechanism is that theoretically paroxysmal activity may start in a healthy brain. A deviation from normal activity of, e.g., organs of the gastro-intestinal or cardio-respiratory systems would lead to an emergence of signals with pathologic frequency composition directed to the central nervous system during sleep, with a possibility to cause epileptic events if these signals happen to be within the resonant ranges of the particular brain circuits. In light of that, it seems reasonable, especially in the pharmacoresistent cases, when no obvious morphological deviations in the brain tissue were found, to consider paying special attention to the visceral state of a patient, and particularly to the visceral systems with clearly rhythmic patterns of activity.

## Author Contributions

All authors listed have made a substantial, direct and intellectual contribution to the work, and approved it for publication.

### Conflict of Interest

The authors declare that the research was conducted in the absence of any commercial or financial relationships that could be construed as a potential conflict of interest.
